# Blood test ordering for unexplained complaints in general practice: the VAMPIRE randomised clinical trial protocol. [ISRCTN55755886]

**DOI:** 10.1186/1471-2296-7-20

**Published:** 2006-03-22

**Authors:** Marloes A van Bokhoven, Hèlen Koch, Trudy van der Weijden, Richard PTM Grol, Patrick JE Bindels, Geert-Jan Dinant

**Affiliations:** 1 University of Maastricht, Care and Public Health Research Institute (CAPHRI), Department of General Practice/Centre for Quality of Care Research, PO Box 616, 6200 MD, Maastricht, The Netherlands; 2 Academic Medical Center - University of Amsterdam, Division of Clinical Methods & Public Health, Department of General Practice, PO Box 22660, 1100 DD, Amsterdam, The Netherlands; 3 University of Maastricht, Care and Public Health Research Institute (CAPHRI), Department of General Practice, PO Box 616, 6200 MD, Maastricht, The Netherlands

## Abstract

**Background:**

General practitioners (GPs) frequently order blood tests when they see patients presenting with unexplained complaints. Due to the low prevalence of serious pathology in general practice, the risk of false-positive test results is relatively high. This may result in unnecessary further testing, leading to unfavourable effects such as patient anxiety, high costs, somatisation and morbidity. A policy of watchful waiting is expected to lower both the number of patients to be tested and the risk of false-positive test results, without missing serious pathology. However, many general practitioners experience barriers when trying to postpone blood testing by watchful waiting. The objectives of this study are (1) to determine the accuracy of blood tests in patients presenting with unexplained complaints in terms of detecting pathology, (2) to determine the accuracy of a watchful waiting strategy and (3) to determine the effects of a quality improvement strategy to promote the postponement of blood test ordering by GPs for patients with unexplained complaints.

**Design:**

General practices are randomised over three groups. Group 1 is instructed to order blood tests immediately, group 2 to apply a watchful waiting policy and group 3 also to postpone testing, but supported by our quality improvement strategy. The trial consists of two sub-studies: a diagnostic study at patient level (group 1 versus groups 2 and 3) and a quality improvement study at GP level (group 2 versus group 3). The diagnostic strategy to be used involves of both customary and innovative tests. The quality improvement strategy consists of two small-group meetings and a practice outreach visit. Patient follow-up ends at 12 months after the initial consultation. Primary outcome measures are the accuracy and added value of blood tests for detecting pathology, the effect of a 4-week postponement of test ordering on the blood test characteristics and the quantity of tests ordered. Secondary outcome measures are the course of complaints, quality of life, satisfaction with care, anxiety of patients and practitioners, determinants of physicians' behaviour, health care utilisation and costs.

**Discussion:**

The innovative aspect of this trial is that it combines a clinical-epidemiological study and a quality of care study.

## Background

### Unexplained complaints

'Unexplained complaints' can be defined as: those complaints for which a general practitioner (GP), after clarifying the reason for encounter, taking history and performing physical examination, is unable to establish a diagnosis.[1] This definition reflects a broad continuum of clinical pictures, ranging from complaints of recent onset to more chronic situations in which the physician is convinced that somatic disease is absent. Newly presented unexplained complaints will in most cases be self-limiting, but they can also develop into chronic complaints, or might be the first sign of serious disease.[2] Since GPs are usually the first health care professionals patients present their complaints to, these complaints belong to the particular expertise of GPs, who are used to deal with this type of complaints autonomously, without referring the patients to hospital.

On average, 13% of consultations involve complaints considered unexplained by the GP.[3] Although only a small minority of these lead to chronicity or serious disease, additional diagnostic testing is often done after history taking and physical examination.[4,5]

### Blood test ordering

It has frequently been suggested that immediate test ordering in unexplained complaints is superfluous.[6-8] Since the pretest probability of serious pathology in patients with unexplained complaints is usually low, the risk of false-positive test results is relatively high. This may result in a chain of unnecessary further testing, which in turn might lead to patient anxiety, high costs, somatisation and a risk of serious side effects or even unnecessary morbidity.[9,10] Applying a watchful waiting policy is recommended because the majority of these complaints are expected to be self-limiting. Patients in whom the complaints are not self-limiting will have a higher prior probability of having serious pathology, and the diagnostic accuracy of tests in this selected group is expected to be higher, because of the lower risk of false-positive test results.

We have found only one guideline on blood test ordering for unexplained complaints in general practice. This guideline, issued by the Dutch College of General Practitioners (NHG), recommends an initial watchful waiting strategy. If a complaint persists, it recommends ordering a limited number of tests (glucose, haemoglobin (Hb), erythrocyte sedimentation rate (ESR) and thyroid stimulating hormone (TSH)).[1,11]

However, these test recommendations are based on theory and consensus rather than on evidence. Moreover, in practice, more blood tests are ordered, or the watchful waiting strategy is not followed. Little is known about the accuracy or additional value of diagnostic blood tests or combinations of such tests, in addition to signs, symptoms and environmental and psychosocial factors, for the purpose of discriminating between self-limiting unexplained complaints and pathology. In addition, the accuracy of some newer tests, such as the carbohydrate deficient transferrin (CDT) test for the detection of pathology in patients with unexplained complaints in general practice, is not yet known.[12] Furthermore, several questions concerning the non-diagnostic effects of test ordering, e.g. on patient and doctor anxiety, remain unanswered. Apart from their diagnostic purposes, GPs frequently order tests for more strategic reasons, e.g. to prevent referral to a specialist or to make the psychosocial nature of complaints more acceptable to the patient, thereby anticipating normal blood test results.[11]

### Improvement of test ordering

Though formal evidence is lacking, one can conclude from the high volume of tests ordered by GPs and the low probability of pathology that there is room for improvement to GPs' blood test ordering behaviour for patients presenting with unexplained complaints.[13,14] It is generally accepted that strategies to improve professionals' behaviour need to be developed systematically, based on barriers to and facilitators of the target behaviour.[15] Determinants of blood test ordering by GPs for patients with unexplained complaints include not only a lack of knowledge about the diagnostic value of blood testing but also practice routines, GPs' tolerance of uncertainty, experienced pressure from patients, tactical motives and the perceived need to reassure patients.[11] This means that a strategy aimed at reducing test ordering by professionals should not only focus on improving diagnostic knowledge but also on skills such as dealing with uncertainty and patient pressure and applying alternative modes to reassure patients. The different types of objectives require adequate and tailored methods of instruction, e.g. teaching a skill requires practising rather than lecturing only. Since patients also seem to play a role in the decision process to order tests, it may be valuable to focus a strategy on patients as well, in order to achieve greater effects.

### Objectives of the study

The first objective of the ongoing study presented here is to determine the accuracy of diagnostic blood tests or combinations of such tests and their value, in addition to signs, symptoms and contextual factors, for the purpose of discriminating between self-limiting diseases and serious pathology in patients presenting with unexplained complaints. The second objective is to compare the accuracy of a watchful waiting strategy with that of immediate test ordering. The third objective is to evaluate the effects of a systematically designed quality improvement strategy for GPs, aiming at the postponement of blood test ordering in patients with unexplained complaints.

### Research questions

1. What is the course of complaints that are considered unexplained by GPs over a period of one year?

2. What is the diagnostic accuracy of blood tests, relative to and in addition to combinations of signs and symptoms, for the purpose of discriminating between self-limiting complaints and early stages of pathology in patients presenting with unexplained complaints?

3. What is the cost-effectiveness of a 4-week watchful waiting policy compared to immediate test ordering in patients presenting with unexplained complaints? 'Costs' in this respect include direct medical costs, absence from work and immaterial costs such as patients' uncertainty, satisfaction and quality of life.

4. What is the cost-effectiveness of a systematically developed strategy to improve GPs' test ordering behaviour, compared to merely instructing them to postpone testing?

### Cooperation

The departments of General Practice of the University of Maastricht and of the Academic Medical Center-University of Amsterdam are cooperating in this study. Data collection ended on December 31, 2004. At the time of writing of this protocol article, we are engaged in data cleaning and analysis.

### Ethical approval and informed consent procedure

#### Ethical approval

The medical ethics committees of both the Academic Medical Center-University of Amsterdam and the University Hospital Maastricht have approved the study.

#### Informed consent procedure

GPs hand out written information and an informed consent form to eligible patients. Patients are given the opportunity to read the information and think about participation before signing the consent form. Patients in group 1 are fully informed about the trial. Patients in groups 2 and 3 are kept naive about the possibility of immediate blood test ordering. This is because it is impossible to blind patients for the test group they are in (immediate or postponed blood test ordering). Bias could be caused by selective dropout of patients and by a Hawthorne effect, as patients can be expected to prefer immediate test ordering over a watchful waiting policy. Patients in groups 2 and 3 are told that the study investigates the way their GPs manage patients with unexplained complaints.

## Design

### Operationalisation of 'unexplained complaints'

Of the complaints that are considered unexplained by GPs according to the definition drawn up by the Dutch College of General Practitioners (NHG), the following 5 were selected: fatigue, abdominal complaints, musculoskeletal complaints, itch and weight changes. These were selected on the basis of the following criteria: commonly seen unexplained complaints in general practice, frequent ordering of blood tests and the possibility that clinically relevant underlying diseases are detected by blood tests.

### Clustered randomised clinical trial

**Figure 1:**
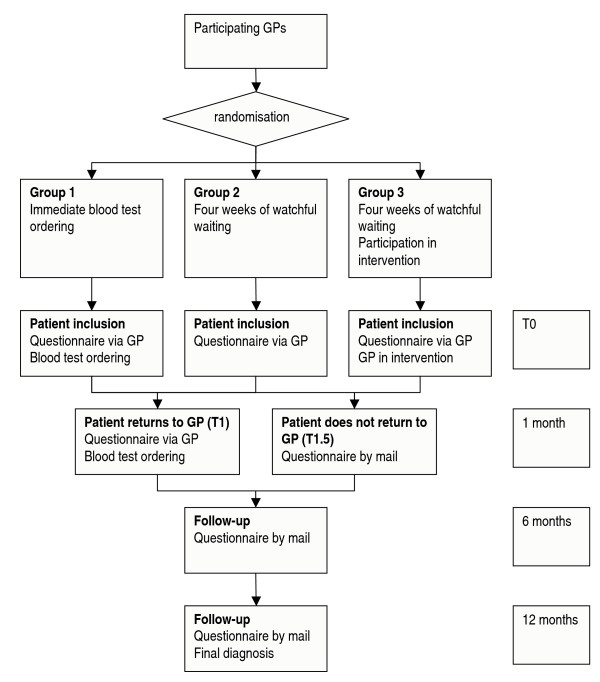
Randomisation scheme.

Participating general practices are randomised over 3 groups (figure 1). GPs in group 1 are instructed to order blood tests immediately at the first consultation. Those in group 2 are instructed to restrict blood test ordering to patients with complaints persisting after four weeks. GPs in group 3 are also instructed to try and postpone test ordering, but they also participate in a quality improvement strategy that supports them in postponing test ordering for patients with unexplained complaints. The study design includes two sub-studies. The first is a diagnostic study comparing patients from group 1 (immediate test ordering) with patients from groups 2 and 3 (4 weeks of watchful waiting). The second is a quality improvement study, comparing group 2 (instruction to postpone test ordering) with group 3 (instruction to postpone test ordering plus quality improvement strategy) in terms of the actual postponement of test ordering.

The reason why we decided to randomise general practices instead of patients or individual GPs is that we assumed there would be contamination, for three reasons. Firstly, it is not possible for GPs to selectively use the communication skills they have learnt during the quality improvement strategy in one patient and fall back to their previous behaviour in the next patient. Secondly, in group practices, patients are sometimes seen by different colleagues at different appointments. If these GPs are in different intervention arms, this may bias the results. Thirdly, patients of different GPs within one practice may exchange experiences about the scientific research project they participate in. The randomisation procedure is carried out separately for the two regions where the participatinjg university departments are located. To achieve allocation concealment, study groups are assigned to GPs by a random number seed computer program that randomises in blocks and is operated by an experienced research assistant.

### Procedure

If, after history taking and physical examination, a patient is considered to have unexplained complaints and gives informed consent, the GP enrols the patient in the study. Depending on the GP's study arm, blood test ordering or a 4-week watchful waiting policy is suggested to the patient. All patients are asked to return to the GP if complaints do not disappear within 4 weeks. If patients return, blood tests are ordered for every patient, irrespective of GPs' study arm. This means that patients in group 1 are then tested for the second time and patients in groups 2 and 3 for the first time.

All tests are performed at the regional laboratory, according to local standard operating procedures and using the local reference values.

Patient follow-up ends at 12 months after the initial consultation.

### Power calculation

Based on previous research, we estimated that approximately 2 percent of patients will eventually be diagnosed with serious pathology.[16] We estimated that 100 patients with pathology will be needed to allow valid conclusions, which means that 5000 patients should be included in the study. Dutch GPs see approximately 500 patients a month, of whom 1–5% are estimated to have unexplained complaints. This means that each GP could on average include 180 patients in one year. These figures mean that we need 27 GPs to participate. Assuming a patient refusal rate of approximately 50 percent, 54 GPs need to be recruited. A total of 5000 patients (approximately 1700 in each study group) would also be sufficient to determine the costs and effects of the quality improvement strategy with enough precision.

### Participants

The project is being carried out in a two regions in the Netherlands, one in the south and one in the west.

#### GPs

For logistic reasons, only GPs associated with certain laboratories for the handling of their test requests can participate in the trial. These laboratories are situated in the western (Haarlem, Almere) and southern (Sittard, Weert, Geldrop, Eindhoven, Helmond, Veldhoven, 's Hertogenbosch) regions. No further GP participation criteria were formulated.

#### Patients

Patients aged 18 years and older, presenting with one of the unexplained complaints mentioned above, who have not consulted their GPs for this complaint in the previous 6 months and who are able to speak, read and write Dutch are eligible to be included in the study. GPs decide to ask patients to participate in the study after history taking and physical examination, so the decision to label the complaints of a patient as unexplained is made entirely by the GPs. Excluded are patients with unexplained complaints for whom the GPs feel that watchful waiting would be unacceptable to them. Patients are asked to participate by their GPs. The GPs are asked to enrol each consecutive eligible patient.

### Blinding

#### Researchers

The researchers are not blinded for the trial group allocation of the participating GPs. They are not involved in the GPs' decision to label patients as having unexplained complaints, nor in the test ordering procedure or the reporting of the results of the laboratory tests.

#### GPs

The GPs are not blinded for the trial group they are randomised to, but they are for the content and format of the quality improvement strategy that aims to support GPs in postponing test ordering. In addition, only those test results from the set of tests we decided to include in the study (see the section entitled 'Diagnostic intervention at patient level' below) they ordered themselves are fed back to them, so they are partially blinded to the test results. Since the effects of test results on the treatment given to patients and on their clinical course are outcome measures of our study, we do not aim at complete blinding to the test results.

#### Patients

Patients in group 1 are fully informed about the blood testing options, whereas patients from groups 2 and 3 are kept naive about the possibility of getting blood tests ordered. All patients are blinded for the possibility that their GP is participating in a quality improvement strategy. In our opinion, full blinding is not possible because there is no placebo for blood testing that is feasible and ethically acceptable.

#### Laboratories

Laboratories are blinded for all patient characteristics except sex and age.

### Diagnostic intervention at patient level

#### Selection of blood tests

Members of an expert panel including GPs and hospital specialists (n = 20) have been individually asked to propose tests which they regarded as useful diagnostic tools in general practice for each of the 5 complaints. All tests mentioned at least twice were included in a complaint-specific set of tests (table 1). If not already included in the set, the four tests recommended in the NHG guideline (glucose, ESR, TSH and Hb) were added. In addition, iron parameters (transferrin saturation (TS) and ferritin), anti-endomysium and carbohydrate deficient transferrin (CDT) were added as indicators of haemochromatosis, celiac disease and alcohol abuse respectively. These three diseases can lead to unexplained complaints, are frequently missed by GPs according to the literature and should be demonstrable with the above blood tests. The diagnostic accuracy of these tests in patients presenting with unexplained complaints in general practice has, however, not yet been established.[12,17-20]

**Table 1:** Sets of laboratory tests per complaint

	**Fatigue**	**Abdominal complaints**	**Musculo- skeletal complaints**	**Weight changes**	**Itch**
Alkaline Phosphatase (AF)	x	x		x	
Alanine aminotransferase (ALAT)	x	x		x	x
Amylase		x		x	
Anti-endomysium		x		x	
Aspartate aminotransferase (ASAT)	x	x		x	
Bilirubin					x
Erythrocyte sedimentation rate (ESR)	x	x	x	x	x
Carbohydrate-deficient transferrin (CDT)	x	x		x	x
Creatinin kinase (CK)			x		
Creatinin	x	x	x	x	x
C-reactive protein (CRP)		x			
Differentiated leukocyte count	x	x		x	x
Eosinophils					x
Ferritin	x	x	x	x	
Gamma glutamyl transferase (GGT)	x	x		x	x
Glucose	x	x	x	x	x
Haemoglobin (Hb)	x	x	x	x	x
Potassium (K)	x		x		
Latex fixation test			x		
Lactate dehydrogenase (LDH)	x			x	
Leukocyte count	x	x		x	x
Monosticon	x				
Total IgE		x			x
Thyroid stimulating hormone (TSH)	x	x	x	x	x
Transferrin saturation (TS)	x	x	x	x	
Urea					x
Uric acid			x		
**Total number of tests**	**17**	**18**	**11**	**17**	**14**

#### Immediate blood test ordering

GPs from group 1 are instructed to order blood tests immediately when including a patient in the study. The GPs are free to decide on the number and type of blood tests. The patient takes the blood test ordering form to the regional laboratory participating in the study. At the laboratory, the tests ordered by the GP are supplemented by the complaint-specific tests from the sets described in table 1. Only results of tests ordered by the GPs themselves are fed back. If a GP does not intend to order any tests (but is obliged to do so by the study protocol), the results of the four tests recommended by the NHG guideline are fed back, because patients expect results after a blood sample has been taken.

#### Watchful waiting for 4 weeks

GPs from groups 2 and 3 propose to the patients they include to observe a 4-week watchful waiting policy. When watchful waiting is not considered feasible by the GP, e.g. because a patient insists on being tested, test ordering is allowed. In that case, the GPs are asked to state the reasons for not postponing test ordering on a special form.

#### Reference standard

The nature of unexplained complaints implies that no proper gold standard exists. Hence, we opted for a delayed type cross-sectional study design.[21,22] In this type of design, the diagnosis after the 12-month follow-up period is used as a reference standard. This diagnosis is established separately by two researchers, making use of information from the GPs' patient records. Differences are discussed and consensus is sought. If consensus cannot be reached, the case is presented to an expert panel that takes the final decision. No restrictions are imposed on GPs as to patient management during follow-up.

### Quality improvement strategy

#### Development of the strategy

Determinants of blood test ordering for unexplained complaints – both those relating to the GPs and those relating to the patients – have been identified previously.[11,23] Based on these determinants, a quality improvement strategy has been developed using a systematic procedure based on intervention mapping techniques.[15] The strategy has been pilot-tested, after which slight adjustments were made based on the pilot results.

#### Content of quality improvement strategy

For each GP in group 3, the strategy consists of two small-group meetings and one outreach visit by the researchers to their practice. Table 2 provides more details on the content of the strategy. The first group meeting is led by a GP experienced in medical diagnostic decision-making and a behavioural scientist experienced in teaching communication skills, while the second meeting is tutored only by the behavioural scientist. The outreach practice visit is made by one of the researchers. We have also developed patient education leaflets and diaries, to be handed out by the GPs, and a video message about watchful waiting, to be shown in the waiting room. All materials have been developed by the research team.

**Table 2:** Elements of the quality improvement strategy

**Elements of strategy**	
Contents of programme	Small group meeting 1 Part 1: Interactive explanation of diagnostic value of diagnostic testing for unexplained complaints and effect of watchful waiting policy on diagnostic value. Part 2: Discussion of difficulties experienced in practice when dealing with patients presenting with unexplained complaints. Goal setting to change behaviour in GPs' own practice. Small group meeting 2 Part 1: Discussion about experiences with behaviour change. Searching for solutions to barriers that have arisen. Part 2: Practicing difficult situations by means of video vignettes. Setting new goals to change own behaviour. Practice visit Discussing individuals' barriers to change and providing suggestions to overcome these, based on stage of change. In between meetings, GPs get the opportunity to work on their goals to change their behaviour.

Materials	– Course book. – Leaflets for patients with information about unexplained complaints. – Diaries about complaints and food intake to hand out to patients to fill in and later discuss together. – Video message for the waiting room, explaining the use of watchful waiting.

#### Procedure

At the beginning of the trial, all GPs in group 3 are invited to the small group meetings, which are organised regionally, usually at the regional hospital. These meetings are held with an interval of approximately four weeks. After these meetings, an appointment is made for the practice visit, which takes place at least 3 months after the second group meeting. GPs are encouraged to prepare for the meetings by doing homework assignments.

#### Control group

GPs in group 2 are the control group for GPs in group 3. In order to ensure a maximum contrast, no strategy is offered to group 2.

### Measurements

All measurement instruments, measurement times and variables are summarised in table 3.

**Table 3:** Instruments and variables.

**Instrument**	**Variables**	**Time points**					**Population**	**Method**
		**T0**	**T1**^#^	**T1.5**^#^	**T6**	**T12**		**- Provision** **- Person who completes questionnaire** **(- Comments)**

GPs' background data	Personal data	x					All GPs	- By mail
	Practice characteristics	x						- GP
	CME	x						
	Laboratory facilities available in practice	x						

Complaint registration form (for each individual type of complaint)	Symptoms	x	x				All patients	- Present in practice
	Signs	x	x					- GP
	Working hypothesis	x	x					
	Degree of unexplainedness	x	x					
	Degree of suspicion of serious pathology	x	x					
	Degree of insecurity of GP	x	x					
	Satisfaction of GP	x	x					

Patients' background data (included in patient questionnaire)	Date of birth	x	x	x	x	x	All patients	- Handed out by GP (T0, T1)
	Country of birth	x	x	x	x	x		- By mail (T1.5-T12)
	Sex	x	x	x	x	x		- Patient
	Marital status	x	x	x	x	x		
	Type of health insurance	x	x	x	x	x		
	Level of education	x	x	x	x	x		

Patient questionnaire	Intensity of complaints	x	x	x	x	x	All patients	- Handed out by GP (T0, T1)
	Course of complaints	x	x	x	x	x		- By mail (T1.5-T12)
	Satisfaction with care	x	x					- Patient
	Anxiety	x	x					
	Quality of life	x	x	x	x	x		
	- RAND 36							
	- Euroqol thermometer							
	Utilisation of health care	x	x	x	x	x		

Test ordering form	Quality of test ordering	x*	x*				All patients	- Present in practice
	Quantity of test ordering	x	x					- GP

Test result form	Test results	x*	x*				All patients	- Laboratory
								- Laboratory staff

Record examination form	Final diagnosis					x	All patients	- Practice/university
	Utilisation of health care					x		- Researcher
	Use of watchful waiting strategy					x		

		**Meeting 1**		**Meeting 2**		**Practice visit**		

Reports of small group meetings	Participation	x		x			All GPs in intervention group	- Meeting room
	Learning effects	x		x				- Researcher

Evaluation forms of small group meetings	Valuation of programme	x		x			All GPs in intervention group	- Meeting room
	Learning effects	x		x				- Researcher
	Suggestion for improvement	x		x				

GP interview in practice	Barriers and facilitators during change					x	All GPs in intervention group	- Practice
	Stages of change					x		- GP

Cost registration	Costs of development and organising of strategy	x		x		x		- University
								- Researcher

* T0 for patients randomised to group 1, T1 for all patients but only if they revisit their GP

^# ^T1 is the time point to measure a follow-up visit by the patient 4 weeks after inclusion. If a patient does not return to the GP, a questionnaire is sent by mail after 6 weeks. This time point is indicated as T1.5.

Most questions in the questionnaires have been formulated by the research team, based on topics found in the literature. Quality of life is measured by the RAND SF36, together with the thermometer of the Euroqol questionnaire. Both are widely used and have been validated extensively.[24-26]

#### Patients

Findings of history taking and physical examination are recorded on a prestructured complaint registration form by the GP after the consultation(s).

All patients are given the first questionnaire at the first consultation and a second questionnaire at the second consultation after 4 weeks. Patients who do not return to their GP after 4 weeks are sent a questionnaire by mail. Patients from all groups receive follow-up questionnaires by mail at 6 and 12 months after the initial consultation.

After 12 months, i.e. at the end of the follow-up period, data on the final diagnosis and health care consumption are collected from the patients' records by the researchers (MB and HK).

Copies of test ordering and result forms are collected by the researchers (MB and HK) from the regional laboratories.

#### GPs

Background data of all participating GPs are collected before the start of the patient inclusion process. Data on whether the GPs are satisfied with the consultation, whether they suspect serious pathology and whether they are certain about the diagnosis are collected from the complaint registration form. The GPs' test ordering behaviour is derived from the test ordering forms and the patient records. GPs in group 3 are asked to complete evaluation forms on process items of the quality improvement strategy. In addition, the determinants of their change processes they mentioned are audiotaped during the practice visit that is part of the quality improvement strategy.

### Data analysis

#### Primary outcome measures of the diagnostic intervention

The first primary outcome is the accuracy and added value of blood tests in detecting serious pathology (per test and in combinations relevant to general practice), related to and in addition to signs and symptoms. Serious pathology is defined as pathology requiring treatment. The second primary outcome is the effect of a 4-week postponement of test ordering on the blood test characteristics.

#### Primary outcome measures of the quality improvement strategy

The primary outcome of the quality improvement strategy is the quantity of tests ordered in relation to the instruction to either order blood tests immediately or to suggest a 4-week watchful waiting policy.

#### Secondary outcome measures

The secondary outcome measures are summarised in table 4.

**Table 4:** Secondary outcome measures

Incidence of unexplained complaints in general practice
Predictive value of GPs' working hypothesis
Duration of unexplained complaints
Effect of unexplained complaints on patients' quality of life
Effect of immediate testing or watchful waiting on patients' satisfaction with care, anxiety, medical consumption and sick leave
Effect of immediate testing or watchful waiting on GPs' satisfaction, anxiety and insecurity
Effect of quality improvement strategy on GPs' knowledge about the value of blood test ordering in unexplained complaints, communication skills and attitudes
Barriers to and facilitators of GPs proposing a watchful waiting strategy
Costs of the quality improvement strategy

#### Types of analysis

An intention-to-postpone analysis will be performed. Longitudinal comparisons will be made using repeated measurements techniques and multilevel analysis to correct for potential clustering of outcomes in GPs and practices. Barriers to and facilitators of change are analysed qualitatively.

#### Non-inclusion analysis

A non-inclusion analysis will be performed to check whether included patients are comparable to patients who are eligible but not included.

## Discussion

### Reasons for publishing this study design

This protocol describes an RCT which combines the generation of new clinical and epidemiological evidence to underpin guidelines on test ordering in unexplained complaints with the implementation of these guidelines. To our knowledge, this is the first RCT to integrate questions on clinical epidemiology and quality of care. The first reason for us to opt for this combination is that it meets suggestions made in the literature to evaluate the value of diagnostic tests just as rigorously as is done with treatments, namely by RCTs, and to pay attention to the implementation of evidence while generating it.[27] Secondly, both sub-studies require large numbers of participants with the same characteristics, and combining the two increases the efficient use of available resources. Apart from the advantages of the combination, however, we also need to mention one methodological disadvantage. Ideally, the evidence on which a quality improvement strategy is based should already be available at the start of development of the strategy, as it might influence its format. In the case of this study, evidence generation on one hand and the design and execution of the strategy on the other run in parallel. Evaluation at the end of the study should clarify whether the new evidence will necessitate alterations in either the diagnostic guidelines or the quality improvement strategy.

General reasons to publish the design of a study before the analyses have begun have been discussed by Godlee and De Bruijn et al.[28,29] In the case of the present study, there was an additional reason to do so. The design of the study presented here is rather complicated because, due to the nature of unexplained complaints, we had to seek methodological solutions that were not always straightforward.[22] By presenting the decisions made in designing this study, we are hoping to start a debate on proper methodology for research into unexplained complaints.

## Competing interests

The author(s) declare that they have no competing interests.

## Authors' contributions

MB participated in working out the protocol, carries out the study in the southern region, will analyse the data of the quality improvement sub-study and wrote the first draft of the manuscript. HK participated in working out the protocol, carries out the study in the western region, will analyse the data of the diagnostic sub-study and helped to draft the manuscript. TW participated in the design of the study, coordinates the quality improvement sub-study and helped to draft the manuscript. RG participated in the design of the study and supervises the quality improvement sub-study. PB is the project leader of the study in the western region, supervises the diagnostic sub-study and helped to draft the manuscript. GJD is the general project supervisor, participated in the design of the study, will supervise the analyses and helped to draft the manuscript. All authors read and approved the final manuscript.

## Pre-publication history

The pre-publication history for this paper can be accessed here:


